# Intimate encapsulation of non-planar electrodes via a viscoplastic interlayer

**DOI:** 10.1093/nsr/nwag297

**Published:** 2026-05-20

**Authors:** Liqian Liu, Xinyue Xiang, Yinglin Zhi, Guoli Chen, Yan Shao, Rui Xia, Daohang Cai, Huiping Wu, Yuda Chen, Jingjia Li, Fuzeng Ren, Shiming Zhang, Chuanfei Guo, Yanhao Yu

**Affiliations:** Department of Materials Science and Engineering, Southern University of Science and Technology, Shenzhen 518055, China; Department of Materials Science and Engineering, Southern University of Science and Technology, Shenzhen 518055, China; Department of Materials Science and Engineering, Southern University of Science and Technology, Shenzhen 518055, China; Department of Materials Science and Engineering, Southern University of Science and Technology, Shenzhen 518055, China; Department of Materials Science and Engineering, Southern University of Science and Technology, Shenzhen 518055, China; Department of Materials Science and Engineering, Yancheng Institute of Technology, Yancheng 224051, China; Department of Materials Science and Engineering, Southern University of Science and Technology, Shenzhen 518055, China; Department of Materials Science and Engineering, Southern University of Science and Technology, Shenzhen 518055, China; Department of Materials Science and Engineering, Southern University of Science and Technology, Shenzhen 518055, China; Department of Materials Science and Engineering, Southern University of Science and Technology, Shenzhen 518055, China; Department of Materials Science and Engineering, Southern University of Science and Technology, Shenzhen 518055, China; Department of Materials Science and Engineering, Southern University of Science and Technology, Shenzhen 518055, China; Department of Electrical and Electronic Engineering, The University of Hong Kong, Hong Kong 999077, China; Department of Materials Science and Engineering, Southern University of Science and Technology, Shenzhen 518055, China; Department of Materials Science and Engineering, Southern University of Science and Technology, Shenzhen 518055, China; Institute of Innovative Materials, Guangdong Provincial Key Laboratory of Sustainable Biomimetic Materials and Green Energy, Southern University of Science and Technology, Shenzhen 518055, China

## Abstract

Implantable electronics often adopt non-planar electrodes to resolve the conflict between conductivity and deformability. Encapsulation of these electrodes becomes a critical challenge for conventional elastic seals due to the elastic-contact-induced interfacial voids and consequential fluid ingress. Here, we present a viscoplastic interlayer that can adapt to the three-dimensional structures of non-planar electrodes, resulting in intimate contact and hermetic encapsulation. This interlayer is a polymeric composite that consists of a long-chain polyisobutylene as the matrix, a short-chain polyisobutylene as the plasticizer, and maleic anhydride-grafted polypropylene as the foreign domains. Its viscoplasticity originates from the chain slippage and permanent disentanglement of the long-chain polyisobutylene, promoted by the plasticizers and confining domains, respectively. When synergized with covalent bonding, the interlayer derives defect-free interfaces between the sealing elastomer and various non-planar electrodes, such as microwires, micropillars, and serpentine electrodes. This intimate encapsulation stabilizes the signal-to-noise ratio of an electromechanical device for 50 weeks in acidic, neutral, and alkaline solutions and extends the *in vivo* duration of signal fidelity for stretchable bioelectronics to a record of 45 weeks. This viscoplastic interlayer provides fruitful implications for improving the long-term stability of implantable bioelectronics.

## Teaser

This work introduces a viscoplastic interlayer, synergized with covalent bonding, to eliminate elastic-contact-induced interfacial voids at the electrode/encapsulation interfaces, achieving a universal intimate encapsulation strategy for non-planar electrodes.

## INTRODUCTION

Implantable electronics offer a transformative approach to advanced healthcare by enabling real-time physiological monitoring [1,2] and targeted therapeutic intervention [3,4] directly at the site of disease. The operation of these electronics relies on encapsulations to prevent biofluid-induced damage [5]. While flexible encapsulations can effectively protect the main units in the short term, the gradual ingress of biofluids along the interface between the electrode and encapsulation hinders the long-term functionality, ranging from reduced signal-to-noise ratios to overall device failures [6]. This phenomenon is particularly evident for the non-planar electrodes (e.g. microwires, micropillars, and serpentine electrodes), which are common designs in implantable electronics for enhanced conformability, biocompatibility, and spatial resolution [7,8].

The ingress of biofluids originates from the leaky contact between the electrode and the encapsulation under strained conditions [9,10]. Contemporary implantable encapsulation materials include polymeric elastomers (e.g. polystyrene block copolymers, polyurethane, and silicones) and plastic or ceramic thin films (e.g. polyimide, parylene C, and silicon nitrides) [11]. As the three-dimensional structure is incompatible with thin film encapsulations, non-planar electrodes count on elastomers for protection. The leaky contact at the electrode/elastomer interface traces back to the entropic elasticity of the elastomers [12]. Upon the tensile deformation induced by internal structure or external loading, the elastic restoring force retracts the polymeric chains, leading to the formation of microscopic voids and the consequential infiltration of fluids. Introducing chemical bonds can mitigate this issue [13], but the elastic contact impedes the bonding density and uniformity, making it challenging to eliminate all defective channels through binding strategies alone. Forming a non-rebound conformal interface that adapts to the three-dimensional geometry becomes a key consideration for preventing the ingression of biofluids [14].

**Figure 1.**
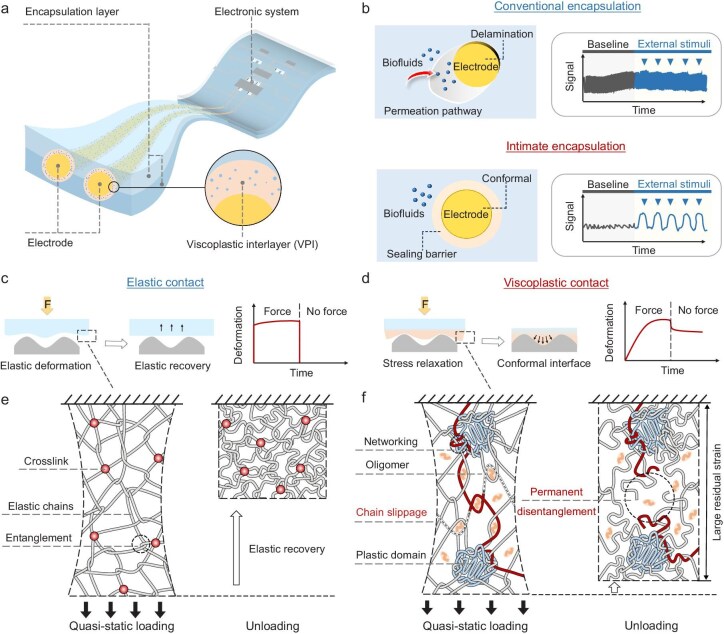
Design principle of the viscoplastic interlayer (VPI). (a) Schematics of the VPI-based encapsulation strategy for the protection of non-planar electrodes. (b) Cross-sectional schematics of the leaky electrode protection under conventional elastic encapsulation and the hermetic electrode protection under the VPI-derived intimate encapsulation. The ingress of biofluid causes a decrease in the signal-to-noise ratio. (c) Schematics of the elastic contact that results in interfacial gaps. (d) Schematics of the viscoplastic contact that leads to conformal interfaces. (e) Schematics of the cross-linked elastomer that undergoes a large degree of elastic recovery after the removal of external loading due to the entropic elasticity. (f) Schematics of the molecular origin of the viscoplasticity in the VPI. The PIB oligomer and MAPP plastic domain induce the slippage and permanent disentanglement of the long-chain PIB network, respectively, leading to energy dissipation and suppressed elastic recovery.

This objective aligns well with the shape-morphing property of viscoplastic materials. A viscoplastic silicone enables morphing electronics to comply with *in vivo* nerve tissue growth while exerting minimal mechanical constraint [15]. A viscoplastic surface effect on polystyrene-based block copolymer derives stretchable seals that hermetically protect planar devices [10]. These two designs cannot directly apply to the encapsulation of non-planar electrodes due to the high intrinsic water permeability of silicones and the limited adaptability of surface effects, respectively [16]. However, they suggest that a bulk layer of viscoplastic, low-permeable rubber would be a straightforward solution for resolving the leaky contact between the encapsulation and non-planar electrode (Fig. 1a–d).

## RESULTS

### Design and characterization of the viscoplastic interlayer

To induce bulk viscoplasticity, the polymer network needs to minimize elastic energy storage and maximize energy dissipation by irreversible mechanisms, such as chain slippage [17], permanent disentanglement [18], and breakage of covalent bonds [19]. Guided by this principle, our viscoplastic interlayer (VPI) was assembled using a physically entangled, lowly permeable polyisobutylene (PIB, Mw∼1 550 000) as the polymer matrix, PIB oligomers (Mw∼400) as the plasticizer, and maleic anhydride-grafted polypropylenes (MAPP) as the foreign plastic domains. The physically entangled PIB matrix had a high tendency to slip its chains under stress due to its non-crosslinking nature (Fig. 1e and f). The PIB oligomers further promoted the chain slippage by increasing free volume and lubricating the long-chain PIB, as shown by the decrease in glass transition temperature and relaxation time of the long-chain PIB (Figs S1 and S2). The MAPP domains ensured the permanent disentanglements through spatially confining the slipped long-chain PIB, which resulted from the partial miscibility of PP in the PIB matrix and the consequent formation of interpenetrating networks between them [20]. This was evidenced by the convergence of the glass transition temperatures of the two components (Fig. S1). The crystallinity of the pristine MAPP and the MAPP in the VPI, characterized by differential scanning calorimetry, was 40.9% and 26.8%, respectively, indicating the disrupted ordering of MAPP chains due to the confinement of the PIB matrix (Fig. S3). Polarized light microscopy further revealed that the PIB chains accumulated around the MAPP domains after stretching, forming a noncrystallizable shell that contributed to the irreversible plastic deformation (Fig. S4). These energy dissipation processes synergistically suppressed the elastic recovery and endowed a large residual strain for the VPI.

**Figure 2.**
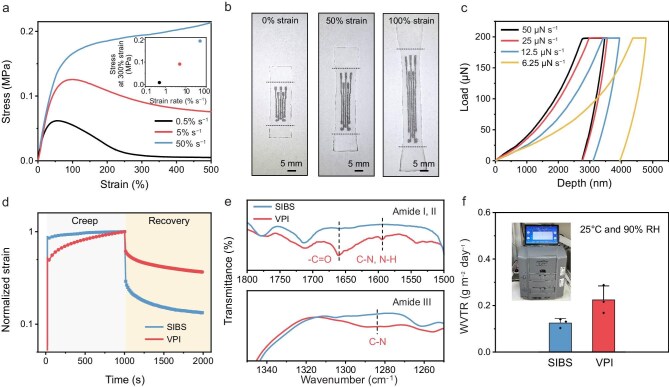
Mechanical and chemical properties of the VPI. (a) Uniaxial tensile stress-strain curves of the VPI film with the mass ratio of 3 : 2 : 1 for the long-chain PIB, PIB oligomer, and MAPP under different strain rates. The inset is the stress at 300% strain under different strain rates. (b) Optical images of the VPI film with 3D-printed liquid-metal electrodes after being stretched to the predefined strains (0%, 50%, and 100%). (c) Nanoindentation load-displacement curves for the VPI film at different loading rates. (d) Creep-recovery tests of the SIBS and VPI films under a constant shear stress of 500 Pa at 1 Hz. (e) FTIR spectra of the SIBS and VPI films attached to the amino-functionalized Au electrodes. (f) WVTR comparison of the SIBS and VPI films with a thickness of 400 µm measured at 25°C and 90% relative humidity. The inset is the measurement equipment, Mocon AQUATRAN Model 3. Independent samples, *n* = 3. Error bars, SD.

The viscoplastic characteristic was evaluated under three deformation modes: tension, compression, and shear. When the mass ratio of the long-chain PIB, PIB oligomer, and MAPP (all ratios in the text refer to this sequence unless noted) was 3 : 2 : 1, the VPI exhibited apparent rate-dependent mechanical responses to tensile strains: strain-softening and strain-hardening effects respectively prevailed at a low strain rate of 0.5% s^−1^ and at a high strain rate of 50% s^−1^, aligning well with the previous observations on viscoplastic materials (Fig. 2a) [15,21]. The stress at 300% strain for 50% s^−1^ reached 0.19 MPa, 23.6 times higher than that of 0.5% s^−1^ (inset in Fig. 2a). This result was caused by the distinct molecular response to the mechanical stress applied under different rates. At low strain rates, the long-chain PIB had sufficient time to disentangle and slip, causing the plastic deformation (the MAPP domains maintained their orientation, Fig. S5). The VPI dissipates elastic energy through viscous flow, leading to viscous yielding. Conversely, the PIB matrix could not relax at high strain rates, transferring the applied stress to the stiffer MAPP domains and resulting in increased strength. The strain-hardening phenomenon nearly disappeared on the fully miscible (PIB matrix/PIB oligomer) and immiscible (PIB matrix/PIB oligomer/SiO_2_) control samples, highlighting the indispensable role of the interpenetrating networks between the PIB matrix and MAPP for achieving the viscoplastic property (Fig. S6). SiO_2_ particles cannot form a continuous stress-transfer network, generating severe interface debonding and microcracks at 100% strain, whereas the VPI interface remains intact (Fig. S7). The VPI film exhibited rapid stress relaxation under a constant strain of 100% (Fig. S8) and retained the degree of deformation after removing the external load, like the plasticine clay (Fig. 2b). The stress relaxation time of VPI films decreased with the increase of the MAPP or PIB oligomer content, varying from 276 to 25 s.

The rate-dependent mechanical property of VPI was also observed under compression induced by nanoindentation (Fig. 2c). The indentation depths under the same load of 200 μN for the loading rates of 50, 25, 12.5, and 6.25 μN s^−1^ were 3472, 3553, 3944, and 4783 nm, respectively. The residual displacement increased from 2757 nm for the 50 μN s^−1^ sample to 3949 nm for the 6.25 μN s^−1^ sample, indicating a high shape-morphing capability of VPI when the loading was slow.

Under a constant shear stress, the creep-recovery test identified a residual strain of 36.7% for the VPI film with a mass ratio of 3 : 2 : 1 (long-chain PIB: PIB oligomer: MAPP, Fig. 2d). The residual strain can be tuned from 24.6% to 45.3% by varying the content of the PIB oligomer and MAPP, suggesting a considerable design space of the viscoplastic property (Fig. S9). In contrast, the residual strain for a conventional elastic encapsulation material—poly(styrene-*block*-isobutylene-*block*-styrene) (SIBS) was only 13.3%. Figure S10 further disclosed the distinct mechanical responses for the elastic SIBS compared to the VPI under tension and compression. The above mechanical measurements, conducted under tension, compression, and shear, consistently verified the viscoplastic nature of the VPI.

In addition to the shape-morphing property, the VPI secures the intimate encapsulation by forming covalent bonds with non-planar electrodes. The maleic anhydride groups in the VPI can undergo a ring-opening reaction to chemically bond with the amino groups on the electrode surface (introduced by surface treatments with 11-aminoundecanethiol hydrochloride molecules). The Fourier transform infrared (FTIR) spectroscopy identified absorption peaks of amide I, II, and III on the VPI-encapsulated electrode, but not on the SIBS-encapsulated electrode (Fig. 2e) [22,23]. This observation confirmed the amide bonds specifically formed at the VPI/electrode interface.

Aside from the tight interfacial contact, the encapsulating materials should maintain a low water vapor transmission rate (WVTR) to prevent bulk permeation [24]. The steady-state WVTR of 400 μm-thick VPI measured at 25°C and 90% relative humidity was 0.22 g m^−2^ day^−1^, about two times higher than that of SIBS under the same film thickness and testing conditions, and orders of magnitude lower than that of silicones and polyurethanes (Fig. 2f) [25,26]. The slightly increased WVTR relevant to PIB was caused by the oligomer-induced increase of free volume [10,27]. No structural defects were observed on the VPI under scanning electron microscopy (SEM) due to the partial miscibility between the PP domain and PIB matrix (Fig. S7b). This feature avoided defect-induced abrupt increases of WVTR commonly observed in encapsulating composites [28].

The VPI film can endure long-term immersion in aqueous solution. Gas chromatography (GC) analysis revealed no characteristic peaks of PIB oligomers after the VPI film was immersed in water at 37°C for 5 weeks, indicating the absence of plasticizer migration (Fig. S11). The viscoplastic characteristics were fully retained after the soaking (Fig. S12).

### Encapsulation of non-planar electrodes

**Figure 3.**
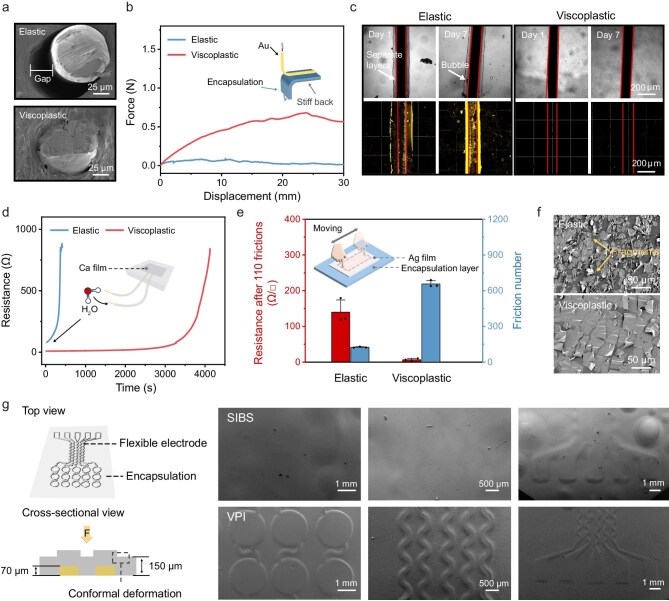
Encapsulation of non-planar electrodes. (a) Cross-sectional SEM images of the Au wire encapsulated by the elastic (SIBS) and viscoplastic (VPI-mediated SIBS) films. (b) The force-displacement curves for the 180° peeling tests of the Au wires on the elastic (SIBS) and viscoplastic (VPI-mediated SIBS) surfaces. The inset is a schematic showing the measurement setup. (c) Fluorescence images of the Au wires encapsulated by the elastic (SIBS) and viscoplastic (VPI-mediated SIBS) films after being immersed in a dye solution for different time periods. (d) Real-time resistance changes of the encapsulated calcium film being immersed in water. The resistance increase is proportional to the water ingress along the interface of the Au wire and the encapsulations made by the elastic (SIBS) and viscoplastic (VPI-mediated SIBS) films. The inset is a schematic showing the measurement setup. (e) The resistance after 110 cycles of friction (left) and the friction number to reach the insulating state (100 MΩ/□, right) for Ag films deposited on the elastic (SIBS) and the viscoplastic (VPI) surfaces. Independent samples, *n* = 3. Error bars, SD. (f) SEM images of the elastic and viscoplastic samples in (e) after 100 cycles of friction. (g) Schematics (left) and corresponding SEM images (right) that compare the shape-adaptive capability of the SIBS and VPI films on serpentine electrodes.

The bulk viscoplasticity, interface bonding, and low WVTR jointly allow VPI to hermetically seal non-planar electrodes, including gold (Au) wires, copper pillars, and copper serpentine electrodes. Au wires are widely used in implantable electronics as interconnects due to their high electrical conductivity, chemical stability, and biocompatibility [29,30]. However, the large aspect ratio and curvature of Au wires pose an integration challenge with conventional elastic encapsulations. As shown in Fig. 3a, a wide gap was formed at the interface when directly hot-pressing the elastic SIBS onto the Au wire surface. Dip-coating 20-μm-thick VPI on the wire surface can fully eliminate the gap between the wire and SIBS, aligning well with the design concept shown in Fig. 1b. The binding strength between the electrode and encapsulation was evaluated by a 180° peel test, which identified a 30 times higher steady-state peel force for the viscoplastic sample compared to the elastic sample (Fig. 3b). This result was caused by the covalent bonds shown in Fig. 2e and the large energy dissipation enabled by the VPI.

The conformal contact and tight binding prevent the ingress of water molecules along the interface of the electrode and encapsulation. When placing the elastically encapsulated Au wire in an aqueous solution containing rhodamine B isothiocyanate (for visualization) with stirring (to induce a fluidic shear force), the confocal fluorescence microscope (CFM) revealed that the elastic contact (Au-SIBS) delaminated and water started to infiltrate on day 1 (Fig. 3c, left). On day 7, a continuous water channel was formed at the interface, as evidenced by the strong fluorescence signal and the intruded bubble. In contrast, no fluorescence signal was observed along the interface between the Au wire and VPI-mediated encapsulation (Au-VPI-SIBS) after 7 days of immersion, demonstrating the high hermeticity and robustness of this interface (Fig. 3c, right).

Suppressing water ingression leads to high electrical stability. We employed the calcium corrosion test to evaluate the electrode stability, as calcium instantaneously changes its resistance upon contact with moisture [9]. The top and bottom surfaces of the calcium film (150 nm in thickness) that was deposited on a polyimide substrate were sealed with a thick SIBS (300 μm) to prevent any resistance change caused by the bulk penetration of water. Two Au wires were attached to the calcium surface, and their extensions out of the calcium film were encapsulated by elastic SIBS or VPI-mediated SIBS. When being immersed in water, the resistance of the viscoplastic group remained stable for over 3500 s, 14 times longer than that of the elastic group (250 s, Fig. 3d).

The anti-friction capability of electrodes can be improved by introducing the VPI design. The transferred silver (Ag) film on the encapsulation layer was repeatedly rubbed by vertical pressure using a rough artificial finger (Fig. 3e). The resistance of the Ag-SIBS sample increased to 140 Ω/□ after 110 cycles of friction and transitioned to an insulating state (100 MΩ/□) after 129 cycles of friction. The resistance of the Ag-VPI-SIBS sample remained steady at 6.6 Ω/□ after 110 cycles of friction and transitioned to an insulating state (100 MΩ/□) until 660 cycles of friction. After 100 cycles of friction, the Ag film of the elastic group was severely damaged, while that of the viscoplastic group maintained interconnected conductive pathways (Fig. 3f). These results validate the enhanced durability of the VPI-mediated thin film electrode against mechanical friction.

The VPI strategy is universally effective in encapsulating non-planar electrodes. The 150-μm-thick VPI can conformally coat microstructural copper electrodes (70 μm in thickness) along their three-dimensional contours (e.g. circular, trapezoidal, and serpentine patterns) via a 90 kPa pressing at 30°C (Fig. 3g). In contrast, the electrode structures became indistinguishable after being encapsulated with an elastic SIBS that had the same film thickness. For pillar electrodes with a height of 200 μm and a diameter of 200 μm, the VPI remained shape-adaptive, while the SIBS created large interfacial gaps due to the elastic rebound (Fig. S13). The interfacial toughness of the VPI-encapsulated Au, Cu, and PAAm electrodes remained steady after cyclic mechanical strain and being immersed in PBS solution (pH ∼7.4, 37°C) for 2 weeks (∼380, ∼100, and ∼50 J m^−2^, respectively, Fig. S14).

### Stabilized signal-to-noise ratio

**Figure 4.**
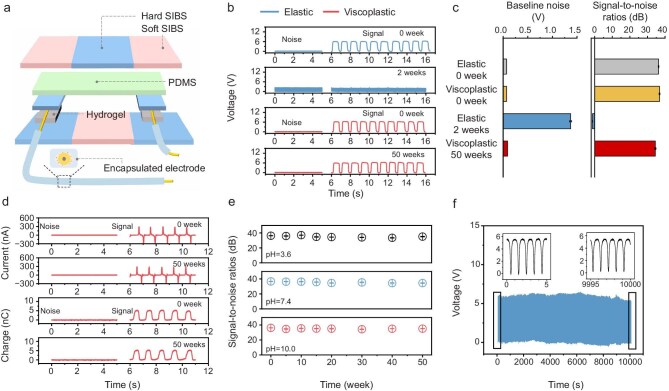
Stable signal-to-noise ratio enabled by the intimate encapsulation of non-planar electrodes. (a) Schematic of the device architecture and the encapsulated Au-wire electrodes. (b) Real-time voltage outputs and corresponding noise levels for the devices with elastically encapsulated (SIBS) and viscoplastically encapsulated (VPI-mediated SIBS) Au wires after being continuously immersed in the PBS solution for different time periods. (c) Quantitative analysis of the baseline noise (left) and signal-to-noise ratios (right) for the voltage outputs of the elastic and viscoplastic groups in (b). (d) Current (top) and charge (bottom) outputs of the viscoplastically encapsulated device before and after 50 weeks of immersion. (e) Signal-to-noise ratios of the viscoplastically encapsulated device after being immersed in acidic (pH ∼3.6), neutral (pH ∼7.4), and alkaline (pH ∼10.0) PBS solutions for 50 weeks. Independent samples, *n* = 3. Error bars, SD. (f) Voltage signal of the viscoplastically encapsulated device undergoing 10 000 cycles of compression after 50 weeks of immersion in neutral PBS solution. The insets depict the voltage output for the device at the initial and final time points of the test.

A stretchable electromechanical device was introduced to prove the concept of the intimate encapsulation of non-planar electrodes in stabilizing the signal-to-noise ratio of bioelectronics. This device was composed of two SIBS packaging substrates, a poly(dimethylsiloxane) (PDMS) contact layer, two SIBS-protected hydrogel electrodes, and two Au wires, operating based on the triboelectric and electrostatic effects driven by periodic contact-separation processes (Fig. 4a and Fig. S15) [31,32]. The Au wires were comparatively encapsulated by the SIBS and VPI-mediated SIBS.

The fresh voltage outputs of the devices with elastically encapsulated Au wires and viscoplastically encapsulated Au wires both reached a peak-to-peak value of 6 V and exhibited a noise level of 0.07 V (without filtering), yielding a signal-to-noise level of 36 dB (Fig. 4b and c). After being immersed in phosphate buffer saline (PBS) solution for 2 weeks, the noise level of the elastic group increased to 1.3 V and fully buried the voltage signal due to the permeation of the solution. The noise originated from the disturbance of the interfacial electrical double layer caused by the penetrated ions. Accordingly, the signal-to-noise level decreased to −1.2 dB. In contrast, the viscoplastic group maintained its voltage output (6 V) and signal-to-noise level (34.5 dB) after 50 weeks of continuous immersion in the PBS solution. The current and charge outputs of the viscoplastic group remained stable at around 270 nA and 5.2 nC throughout the 50 weeks, respectively (Fig. 4d). The signal-to-noise ratios for the current and charge measurements were steady at 26.6 dB and 33.8 dB, respectively, highlighting the effectiveness of the VPI in maintaining a high signal-to-noise ratio (Fig. S16).

The VPI-mediated electrode protection can endure acidic and alkaline environments. The signal-to-noise ratios for the voltage outputs of the viscoplastic group immersed in the PBS solutions with pH values of 3.6, 7.4, and 10.0 all remained stable for 50 weeks (Fig. 4e). After consecutively being immersed in the PBS solution for 50 weeks and cyclically compressed 10 000 times, the voltage output of the viscoplastic group was steady at around 5.7 V (Fig. 4f). These results confirmed the high chemical and mechanical stability of the VPI-mediated electrode protection.

### Long-term *in vivo* operation

**Figure 5.**
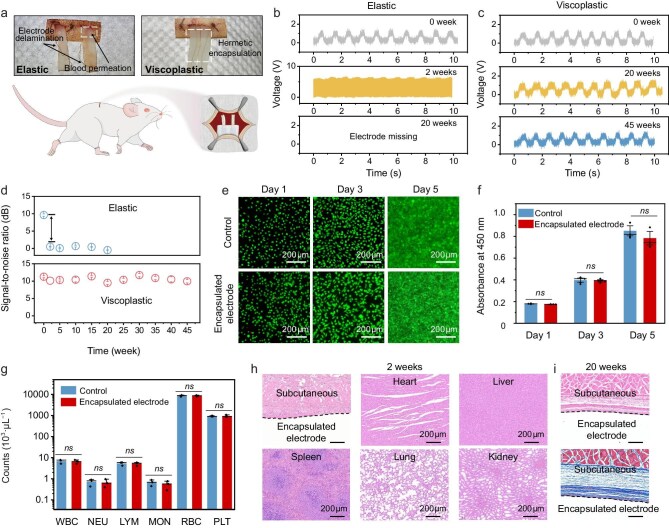
Long-term *in vivo* operation of bioelectronics enabled by the intimate encapsulation of non-planar electrodes. (a) Schematics (bottom) for the subcutaneous implantation of the device shown in Fig. 4 in the dorsal region of a rat and corresponding photographs (top) of the elastically (SIBS) and viscoplastically (VPI-mediated SIBS) encapsulated electrodes after 10 weeks of implantation. (b and c) *In vivo* voltage outputs of the elastic (b) and viscoplastic (c) groups at different time points post-implantation. (d) Signal-to-noise ratios for the elastic and viscoplastic groups during a 45-week implanted period. (e) Live/dead staining of L929 cells co-cultured with the viscoplastically encapsulated electrode over the course of 5 days, showing comparable cell density and morphology to the control group. (f) Quantitative cell viability analysis of the L929 cells for 1, 3, and 5 days. Independent samples, *n* = 5. Error bars, SD. (g) Analysis of complete blood counts for the healthy rats (control) and rats implanted by the device with viscoplastically encapsulated electrodes for 2 weeks. The main detection indicators were white blood cell (WBC), neutrophil (NEU), lymphocyte (LYM), monocyte percentage (MON), red blood cell (RBC), and platelet (PLT). Independent samples, *n* = 3. Error bars, SD. (h) H&E staining of major organs (heart, liver, spleen, lung, kidney) and subcutaneous tissues surrounding the encapsulated electrodes after 2 weeks of implantation. (i) Histological analysis of hypodermis after 20 weeks of implantation: H&E staining (top) shows the tissue structure, and Masson’s trichrome staining (bottom) indicates the collagen deposition. Scale bar: 200 μm; ns: not significant.

The intimate electrode encapsulation enables a substantially extended lifespan for the implanted bioelectronics. After the implantation of the electromechanical device shown in Fig. 4a in the dorsal region of a rat for 10 weeks, severe delamination was observed between the Au wire and the encapsulation in the elastic group (Au-SIBS, Fig. 5a and Fig. S17). This delamination caused the permeation of biofluids into the device through the open volume at the electrode/encapsulation interface. The electrode encapsulation in the viscoplastic group remained hermetic, and no sign of leakage was spotted after 45 weeks of implantation. The *in vivo* voltage output of the device with the elastically encapsulated electrodes reached 1.3 V at day 1. However, this voltage signal was replaced by the baseline noise after 2 weeks of implantation (Movie S1), and the electrodes disappeared after 20 weeks of implantation (Fig. 5b) due to the delamination and blood permeation observed in Fig. 5a. The device with the viscoplastically encapsulated electrodes delivered a voltage output of 1.3 V on day 1. This output remained steady after 45 weeks of implantation (Fig. 5c and Movie S2). The signal-to-noise ratio of the elastic group decreased from 9.6 to 0.5 dB after 2 weeks of implantation, while that of the viscoplastic group was stable at around 10.0 dB throughout the 45 weeks of implantation, the longest *in vivo* stable-recording time reported to the best of our knowledge (Fig. 5d and Table S1).

The VPI-based electrode encapsulation was biocompatible. The live cell density of the encapsulated electrode was comparable to that of the blank group during a 5-day culturing period (Fig. 5e). The L929 mouse fibroblast cells exhibited a typical elongated morphology with reasonable distributions (Fig. S18). No statistically significant difference in cell viability was observed between the viscoplastically encapsulated electrode and the control group after 5 days of culture in the cytotoxicity assays (Fig. 5f). The blood routine parameters (e.g. WBC, NEU, LYM, MON, RBC, PLT) for the electrode group were comparable to the control group after 2 weeks and 20 weeks of implantation (Fig. 5g and Fig. S19). No inflammatory response was induced in the subcutaneous tissues surrounding the encapsulated electrode or in major organs (heart, liver, spleen, lungs, and kidneys) after 2 weeks and 20 weeks of implantation (Fig. 5h and i). These results verified the high biosafety of this encapsulation strategy.

## DISCUSSION

We have developed a viscoplastic interlayer to enable intimate encapsulations of non-planar electrodes, including microwires, micropillars, and serpentine electrodes. The viscoplasticity was made possible by inducing permanent slippages and disentanglements of long-chain PIB network through short-chain PIB plasticizers and MAPP domains. This interlayer has high shape-morphing capability and can covalently bond to foreign metal surfaces, leading to conformal contacts with non-planar electrodes and thorough elimination of interfacial voids. The resulting encapsulation addressed the issue of fluid ingress commonly observed at the interface between conventional encapsulation and three-dimensional electrodes, stabilizing the signal-to-noise ratio of an electromechanical device at around 35 dB for 50 weeks in the PBS solution. This design accordingly improved the *in vivo* lifespan of the device from less than 2 weeks to more than 45 weeks, providing a general strategy for hermetic sealing of three-dimensional biomedical electrodes. For stretchable applications, the viscoplastic encapsulation limits elastic recovery and introduces temperature sensitivity. This shortcoming can be mitigated by finely tuning the ratio of elasticity, viscosity, and plasticity, or spatially designing the mechanical properties of the encapsulation.

## MATERIALS AND METHODS

All experimental details and methods are provided in the Supplementary data.

## Supplementary Material

nwag297_Supplemental_Files
