# Supplementary material for: Intimate encapsulation of non-planar electrodes via a viscoplastic interlayer
Source: Natl Sci Rev. 2026 May 20;13(12):nwag297. doi: 10.1093/nsr/nwag297 (PMC13325494; doi:10.1093/nsr/nwag297)
Supplement: nwag297_Supplemental_Files [file nwag297_supplemental_files.zip › Supplmentary data.pdf]

Supplementary Information for

Intimate encapsulation of non-planar electrodes via a viscoplastic interlayer

Liqian Liu[1,†], Xinyue Xiang[1,†], Yinglin Zhi[1], Guoli Chen[1], Yan Shao[1,2], Rui Xia[1], Daohang Cai[1], Huiping Wu[1], Yuda Chen[1], Jingjia Li[1], Fuzeng Ren[1], Shiming Zhang[3], Chuanfei Guo[1] and Yanhao Yu[1,4,*]

[1]Department of Materials Science and Engineering, Southern University of Science and Technology, Shenzhen 518055, China;

[2]Department of Materials Science and Engineering, Yancheng Institute of Technology, Yancheng 224051, China;

[3]Department of Electrical and Electronic Engineering, The University of Hong Kong, Hong Kong, China;

[4]Institute of Innovative Materials, Guangdong Provincial Key Laboratory of Sustainable Biomimetic Materials and Green Energy, Southern University of Science and Technology, Shenzhen 518055, China

***Corresponding author.** E-mail: yuyh@sustech.edu.cn

[†]Equally contributed to this work.

MATERIALS AND METHODS

Materials

Polyisobutylene (PIB) with an average molecular weight (Mw) of 1550000, 11-aminoundecanethiol hydrochloride (AUT), (3-aminopropyl) triethoxysilane (APTES), and ammonium persulfate (APS) were purchased from Aladdin Co., Ltd. Polypropylene grafted by 8-10 wt% maleic anhydride (MAPP) with an average molecular weight of 9100, and toluene were purchased from Sigma-Aldrich Co., Ltd. PIB oligomer with an average molecular weight of 400 was supplied by Korean Daelim Co., Ltd. Poly (styrene-block-isobutylene-block-Styrene) (SIBS, SIBSTAR 102T) with 15 wt% of polystyrene and poly (styrene-block-isobutylene-block-Styrene) (SIBS, SIBSTAR 103T) with 30 wt% of polystyrene were purchased from Kaneka Co., Ltd. Poly(dimethylsiloxane) (PDMS, Sylgard 184) was purchased from Dow Corning. SiO_2 spheres (5 μm) was purchased from Yumu New Material Co., Ltd. Acrylamide (AAm) and N, N′-methylenebisacrylamide (MBAA) were purchased from Sigma-Aldrich Co., Ltd. Sodium chloride (NaCl) and rhodamine B isothiocyanate (RITC) were purchased from Shanghai Macklin Biochemical Co., Ltd. Eagle's minimum essential medium (EMEM), fetal bovine serum (FBS), and penicillin-streptomycin solution (P/S) were purchased from Thermo Fisher Scientific Inc. L929 mouse fibroblast cells were purchased from BeNa Culture Collection Co., Ltd. Cell counting kit-8, calcein AM, propidium iodide, 4'-6-diamidino-2-phenylindole (DAPI), and phosphate-buffered saline (PBS) solution were purchased from Shanghai Beyotime Biotechnology Co., Ltd. FITC Phalloidin was purchased from Shanghai Yeasen Biotechnology Co., Ltd. The UV adhesive was purchased from Norland Products Inc.

Fabrication of VPI Films

VPI films with a mass ratio of 3:2:1 (high MW PIB: low MW PIB: MAPP) were prepared as follows. First, 3 g of high MW PIB, 2 g of low MW PIB, and 1 g of MAPP were added to 60 mL of toluene. The mixture was then stirred at 125°C under an oil bath with a water-cooled reflux condenser for 2 hours until the MAPP was completely dissolved. Subsequently, the temperature was reduced to 85°C, and stirring was continued for an additional 3 hours to ensure thorough mixing of the components. After the solution cooled to room temperature, it was cast

into a glass petri dish treated with oxygen plasma. To control the solvent evaporation rate, the dish was first sealed with aluminum foil and left undisturbed for 12 hours, followed by puncturing small holes in the foil to allow slow toluene evaporation. Finally, the film was placed in a vacuum oven at 50°C for 12 hours to remove the residual solvent. To systematically investigate the compositional influence, a series of VPI films with different mass ratios were also prepared by varying the proportions of high MW PIB, low MW PIB, and MAPP (3:1:1, 3:3:1, 3:1:2, and 3:1:3).

Structure and property characterizations of VPI Films

Dynamic mechanical analysis (DMA): DMA was performed on a dynamic mechanical analyzer (Discovery DMA 850, USA) operating in single-cantilever mode with a temperature range of -90°C to 160°C, with a frequency of 1 Hz and a heating rate of 5°C min^{-1}.

Differential scanning calorimetry (DSC): The crystallinity of the samples was assessed by Netzsch DSC214 differential scanning calorimetry. Samples (~3 mg) were placed in an aluminum-sealed crucible and heated from 50 to 220°C at 20°C min^{-1}. The percent crystallinity was derived by normalizing the sample's melting enthalpy to the reference enthalpy of 100% crystalline MAPP (207 J g^{-1}).

Polarized Light Optical Microscopy (PLOM): Samples were observed by PLOM using a Leica DM 4P microscope equipped with polarizers.

Wide-angle X-ray scattering (WAXS): The experiment was performed using the HomeLab system (Rigaku, Tokyo, Japan). The rectangular beam had dimensions of 100 x 100 μm^2, and the wavelength of the light was 0.154 nm. All measurements were carried out with an exposure time of 10 s.

Mechanical measurements: Stress-strain curves were obtained using an electromechanical universal testing machine (Model CMT6203). To investigate the mechanical response of the material at different strain rates, tests were conducted at 0.05% s^{-1}, 0.5% s^{-1}, 5% s^{-1}, and 50% s^{-1}. Samples were prepared as rectangular strips with a length of 10 mm and a width of 8 mm. For stress relaxation experiments, the samples were first stretched to 100% strain at a rate of 50% s^{-1}, followed by holding the strain constant for 60 min to characterize the stress relaxation

behavior.

Gas chromatography (GC): Accelerated aging studies were conducted by immersing VPI films (400 μm in thickness) in water at 37°C. The immersion medium was collected and analyzed by gas chromatography to detect the release of PIB oligomers. The analysis was performed using an Agilent 7890B-5977A gas chromatography-mass spectrometry instrument with tetrahydrofuran as the solvent. The injection volume was 1 μL, the split ratio was 10:1, and the column temperature range was 60 to 300°C at a rate of 10°C min^{-1}.

Nanoindentation characterization: Nanoindentation was performed using a Hysitron TI950 nanoindenter (Bruker, USA) with a Berkovich diamond tip. Constant strain rate-controlled tests were conducted in load-controlled mode. Each indentation test was repeated five times, with adjacent indentation points separated by at least 10 μm. In the holding phase, the maximum load was set to 200 μN, and the peak holding time to 60 s. In the loading/unloading phase, different loading/unloading rates (6.25, 12.5, 25, and 50 μN s^{-1}) were achieved by setting corresponding loading/unloading times of 32, 16, 8, and 4 s, respectively. Load-displacement (L-D) curves were recorded throughout the tests.

Rheological measurements: Creep-recovery tests were conducted on the HAAKE MARS 40 type rheometer (ThermoFisher Scientific). Samples were prepared as circular discs (diameter: 20 mm) to match the plate diameter. The tests were performed under a constant shear stress of 500 Pa, with a creep period of 1,000 s followed by a recovery period of 1,000 s. The plasticity of the VPI samples was quantified using the following formula: the degree of plasticity = $\frac{\varepsilon_i}{\varepsilon_{max}}$, where ε_i is the irreversible strain after recovery and ε_{max} is the maximum strain at the end of the creep test.

Fourier transform infrared spectroscopy (FTIR): FTIR spectra were acquired using a PerkinElmer spectrometer (Spotlight I2000i) in the wavenumber range from 4000 to 400 cm^{-1}.

Water vapor transmission rate (WVTR): The WVTR was measured using a moisture permeation analyzer (Mocon, AQUATRAN Model 3). The tests were conducted at 25°C and 90% relative humidity (RH) with a round area of 5.64 cm^2. The films (5 cm × 6 cm) with a thickness of 400 μm were placed in the testing chamber, allowing water vapor flow on one side

and dry nitrogen carrier gas purging on the opposite side. The water vapor permeating through the sample was carried by the nitrogen gas and transported to the water vapor sensor for real-time detection and recording.

Scanning electron microscopy (SEM): SEM images were acquired with a FE-SEM TESCAN MIRA3 in SE mode at an accelerating voltage of 5 kV. Prior to imaging, the samples were coated with a thin Pt layer by magnetron sputtering.

Fabrication and characterization of encapsulated non-planar electrodes

Fabrication of elastic/viscoplastic-encapsulated electrodes: For the SIBS encapsulation layer, 20 g of SIBS was dissolved in 100 mL of toluene under continuous stirring at room temperature until complete dissolution was achieved. SIBS films with a thickness of 200 μm were prepared using the same solution-casting method used for the VPI films. For the elastic group, Au wire was encapsulated between two SIBS films by hot-pressing at 140°C for 10 min. For the viscoplastic group, the Au wire encapsulation process proceeded as follows. The Au wire was first immersed in a 1 mM ethanolic solution of 11-aminoundecanethiol hydrochloride (AUT) for 12 hours to form self-assembled monolayers (SAMs) on the surface. Then the Au wire was immersed in the VPI precursor solution and maintained at 60°C in an oven for 5 hours. The Au wire was withdrawn vertically, resulting in a uniform liquid film on the surfaces. The toluene solvent was then allowed to evaporate at ambient conditions, ultimately forming a continuous VPI film on the electrode. A sandwich structure was constructed by placing the VPI-coated Au wire between the SIBS films. The electrode was sealed through hot pressing at 140°C for 10 min. For the Cu and polyacrylamide (PAAm) hydrogel thin-film electrodes, they were subsequently immersed in an ethanol/water (9:1, v/v) solution containing 5 wt% APTES for 2 h, followed by immersion in the VPI precursor solution at room temperature for 5 h. For cylindrical Cu electrode array encapsulation, SIBS (elastic group) was attached to the electrode surfaces and hot-pressed at 30°C for 10 min. In the viscoplastic group, the preprocessed electrode array formed a VPI interlayer via dip-coating and was subsequently sealed with SIBS via hot-pressing. The serpentine electrodes were fabricated on a copper-coated polyimide (PI) film using a laser cutter (WE-6040, Beyond Laser Co., Ltd.) according to the predefined pattern. The polymer film (SIBS or VPI) with a thickness of 150 μm was mounted onto the electrode

surface and subjected to hot-pressing at 30°C for 10 min. The embedding of the electrode array into the substrate surface was examined by SEM.

Interfacial toughness characterization: For the 180° peel test, the pristine and treated electrodes were mounted onto the surface of an SIBS film (300 μm in thickness) through the encapsulation process. The test was conducted at a constant peeling speed of 10 mm min^{-1}. To verify the stability of interfacial adhesion under mechanical strain, the encapsulated electrodes were subjected to cyclic mechanical strain testing: Au (bending radius of 10 mm, 1000 cycles, 1 Hz), Cu (bending radius of 5 mm, 10,000 cycles, 1 Hz), and PAAm (stretching at 30% strain, 10,000 cycles, 1 Hz). To test the integrity of the interface in an aqueous environment, the Au and Cu electrodes were immersed in PBS solution (pH ~7.4, 37°C) for 2 weeks.

Permeation test: To investigate the penetration of water molecules at the interface, an aqueous solution containing RITC molecules was prepared. The encapsulated electrodes were immersed in the fluorescent solution and continuously stirred at 100 rpm at room temperature to simulate fluid shear forces. Bright-field and confocal fluorescence images of the electrodes were acquired using a Zeiss LSM 980 instrument after 1 day and 7 days of immersion.

Calcium corrosion test: A 150 nm-thick calcium layer was deposited on a 50 μm-thick polyimide film through thermal evaporation and subsequently transferred to a 300 μm-thick SIBS film surface. Two pre-encapsulated Au wires were connected to the calcium film as electrodes, followed by covering with another SIBS layer and sealing through edge hot-pressing. The assembled device was immersed in an aqueous solution for real-time monitoring of electrical performance using a DAQ6510 Data Acquisition Multimeter System (Keithley Instruments).

Friction test: A 300 nm thick silver (Ag) film was deposited on a glass substrate by thermal evaporation. The Ag film (10 mm × 20 mm) was faced against the SIBS/VPI surface and gently pressed by a flat glass plate, transferring the electrode to the substrate. The sample was secured to the platform using cyanoacrylate adhesive (Krazy Glue, 3M CA40H) to maintain the test stability. Copper foil tabs were attached to both ends of the Ag film as electrode leads. The friction object was prepared by mixing PDMS base and curing agent at a 3:1 mass ratio. A

fingerprint was imprinted into the half-cured state PDMS to ensure surface roughness. The sample was then rubbed with the textured PDMS at a speed of 20 mm s^{-1}. Resistance changes during cyclic friction were monitored using a Keithley DAQ6510 system until the electrode transitioned from conductive to an insulating state.

Electrode encapsulation for bioelectronic devices

Fabrication of the electromechanical devices: The bioelectronic device features a vertically stacked structure comprising a SIBS packaging substrate (triboelectric layer), a PDMS layer (triboelectric layer), a SIBS-encapsulated polyacrylamide hydrogel (electrode layer), and the elastic- or viscoplastic-encapsulated Au lead. For the hard SIBS, 10 g of SIBS (30 wt% PS) was dissolved in 40 mL of toluene. For the soft SIBS, 10 g of SIBS (15 wt% PS) and 2.5 g of low MW PIB were dissolved in 40 mL of toluene. Both solutions were stirred at room temperature for 5 h to form a homogeneous solution. Then, 1.5 mL of the hard SIBS precursor solution was cast into a rectangular mold (25 mm × 20 mm). When hard SIBS was in a semi-cured state, it was removed from both end regions (10 mm × 20 mm), and 1 mL of the soft SIBS precursor solution was filled in the blank areas. Mutual diffusion of toluene during the film solidification yielded a seamless soft–hard SIBS composite substrate. The PDMS film was prepared by mixing the silicone base and curing agent at a 10:1 mass ratio. 1 mL of the PDMS precursor solution was cast into a rectangular mold (15 mm × 10 mm) and cured in an oven at 80°C for 4 hours. The PDMS film was treated with oxygen plasma (300 W, 5 min), and then immersed in an ethanol/water mixture (9:1 v/v) containing 5 wt% APTES for 2 hours, and finally dried at 80°C for 1 hour. An adhesive interlayer was prepared by dissolving 20 g of SIBS and 0.5 g of MAPP in 100 mL of toluene, followed by stirring at 125°C under reflux for 4 h. The resulting SIBS/MAPP solution was spin-coated onto the PDMS substrate as an adhesive layer. Stable adhesion was achieved by placing a 200 g weight on the PDMS/SIBS in an oven at 80°C for 4h. The conductive PAAm hydrogel was synthesized by dissolving 0.2 g of AAm (monomer), 1 μL of 0.1M MBAA (crosslinker), 20 μL of 0.1 M APS (initiator), and 0.01 g of NaCl in 1 mL of deionized water. The mixture was cured under 365 nm UV light in a vacuum glove box for 10 min. The encapsulated Au leads were placed beneath the PAAm hydrogel and covered with hard SIBS solution. The hard SIBS was cured to seal the assembly and prevent water

evaporation. Final device integration was achieved through edge sealing via hot-pressing at 140°C.

Electrical measurement of the device: The electro-mechanical devices with elastic- or viscoplastic-encapsulated electrodes were immersed in neutral PBS solution. The voltage output was measured by a Keithley 6514 electrometer at weeks 0, 2, and 50. For devices with viscoplastic encapsulation, current and charge outputs were additionally recorded. The pH stability of viscoplastic-encapsulated devices was further assessed by immersion in PBS solutions at pH 3.6, 7.4, and 10.0 for 50 weeks. Output voltage measurements were performed every five weeks throughout the immersion period. To verify long-term stability in the in vitro physiological environment, the output performance under cyclic compression was evaluated after 50 weeks of immersion. Devices were fixed on a stationary platform using double-sided adhesive tape and subjected to 10,000 compression cycles at 1 Hz frequency using a linear actuator (P01-37×120-C_C1, Linmot). The electrometer recorded the output voltage during mechanical cycling.

Implantation and measurement of the device: All experimental animal procedures and protocols were approved by the Animal Care and Use Committee of Southern University of Science and Technology (Protocol Number: SUSTech-JY202503131). The in vivo study was performed on healthy male Sprague-Dawley rats (6-8 weeks old, 200-250 g). The electro-mechanical coupling bioelectronic devices (25 mm × 20 mm) were sterilized with 75% ethanol and UV irradiation before experimentation. General anesthesia was induced and maintained with isoflurane vapor (1-3%). The anesthetized rat was fixed in the prone position, and the dorsal surgical area was shaved and disinfected with povidone iodine. A 15 mm cutaneous incision was made along the dorsal midline using surgical scissors. The subcutaneous tissue was bluntly dissected to create a pocket for the device. The incision was then sutured closed in layers using 4-0 taper-tipped PGA absorbable sutures. The output signals were recorded using an electrometer (Keithley 6514) connected to the exposed Au leads to verify the functionality of the device. At last, the rats were allowed to recover from general anesthesia and return to the normal breeding environment. For long-term implantation, voltage outputs of the device were recorded every four weeks. All animals were euthanized by CO_2 inhalation at the study endpoint.

Calculation of signal-to-noise ratio: At each measurement time point, at least five consecutive cycles of the operating signal were acquired. The signal-to-noise ratio (SNR) is defined as the ratio of the signal power to the noise power, using the following formula:

$$\mathrm{X} = \sqrt{\frac{1}{N}\sum_{n=0}^{N}(A_n)^2}$$

$$\mathrm{SNR} = 20\log_{10}\frac{X_{signal}}{X_{noise}}$$

where A_n denotes the amplitude at the n-th sampling point, and X_{signal} and X_{noise} denote the root-mean-square (RMS) amplitude of the signal and noise, respectively.

Biocompatibility assessment

Cell cytotoxicity: L929 mouse fibroblast cells were cultured in EMEM medium supplemented with 10 vol% fetal bovine serum and 1 vol% penicillin-streptomycin. The cells were incubated at 37°C with 5% CO_2 in a humidified atmosphere incubator. The cytocompatibility of the encapsulated electrode was assessed using a CCK-8 assay with the L929 cell line. The samples were previously washed with 75% ethanol and sterilized by UV for 30 min. L929 cells were seeded in 48-well plates at a density of 2×10^4 cells per well. The sterilized and blank (growth media alone) samples served as substrates for cell growth. Cells were incubated for 1, 3, and 5 days, and the culture medium was changed every day. At each time point, the medium was replaced with fresh medium containing 10 vol% CCK-8 solution. After 1 hour of incubation, the reaction solutions were transferred to a 96-well plate. Absorbance at 450 nm (n = 3) was measured using a microplate reader (Synergy H1, BioTek Instruments, Inc.) to assess cell viability.

Cell live/dead staining assay: The cell culture and preparation steps followed the same protocol as the CCK-8 assay. Cells were seeded in 48-well culture dishes (2×10^4 cells well^{-1}) containing 500 μL cell culture medium and cultured for 1, 3, and 5 days. The staining solution was prepared by mixing calcein AM, propidium iodide, and PBS solution at a 1:1:1000 volume ratio. The cytoskeleton and nucleus were stained with calcein-AM (green, ex: 494 nm) and propidium iodide (red, ex: 535 nm), respectively. After incubation for a certain time, the culture

medium was replaced with 200 μL of staining solution and re-incubated at 37°C for 15 min. The cells were then gently rinsed with PBS to remove excess dye. Fluorescent images were obtained using a confocal fluorescence microscope (Leica DMI6000 B).

Cell morphology examination: L929 cells were seeded in 48-well plates at 2×10^4 cells per well and cultured for 24h. The cells were treated according to the experimental protocol of the CCK-8 assay, except for the staining part. The cytoskeleton and nucleus were stained with FITC phalloidin (green, ex: 495 nm) and DAPI (blue, ex: 340 nm), respectively. The staining procedure was performed as follows: First, the old cell culture medium was discarded, and the cells were washed twice with PBS buffer. The cells were then fixed with 4% paraformaldehyde for 15 min and rinsed twice with PBS. Subsequently, cells were permeabilized with 0.1% Triton X-100 for 15 min and washed twice with PBS. They were later stained with FITC phalloidin for 30 min at room temperature, then rinsed twice with PBS. Nuclei were counterstained with DAPI for 5 min, then rinsed twice with PBS. The stained cells were visualized by the laser scanning confocal microscope (Leica TCS SP8 X).

Histology assay: The biocompatibility of the electro-mechanical coupling bioelectronic devices was histologically evaluated. The rats were euthanized at 2- and 20-week post-implantation. The implant device with adjacent tissue and major organs (heart, liver, spleen, lung, and kidney) were collected and fixed in 4% paraformaldehyde overnight. Tissues were then dehydrated through a graded ethanol series, cleared in xylene, and embedded in paraffin blocks. Serial sections of 5 μm thickness were cut using the Leica RM2016 Cryostat (Leica, Germany). For morphological analysis, sections were stained with hematoxylin and eosin (H&E) and Masson's trichrome. All samples were observed under a light microscope.

Complete blood count (CBC): To determine the physiological and biochemical states of the animals, the blood and serum tests were performed at 2- and 20-week post-implantation. The blood samples were collected from the orbital venous plexus in sedated animals. For each sample, 0.5 mL of blood was placed in an EDTA tube and analyzed by a 5-part Auto Hematology Analyzer (DF52Vet, Dymind, China).

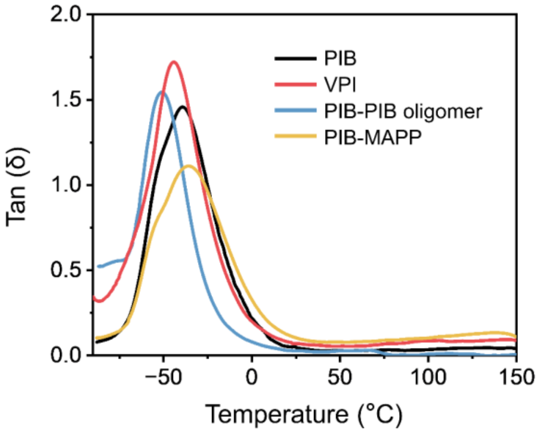

Supplementary Figure 1. DMA tan δ plot as a function of temperature for PIB, VPI, PIB/PIB oligomer, and PIB/MAPP.

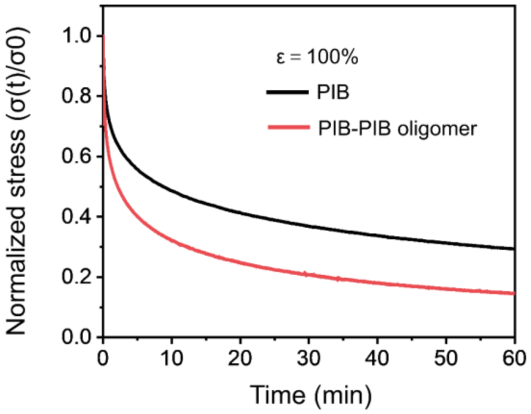

Supplementary Figure 2. Stress relaxation curves of PIB and PIB matrix/PIB oligomer (3:2) films were primarily stretched to 100 % strain and then allowed to relax for 60 min.

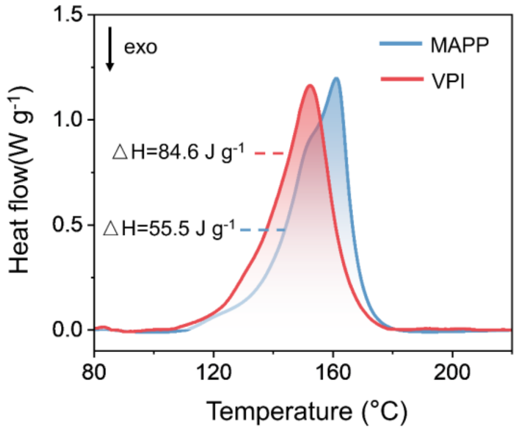

Supplementary Figure 3. DSC curves of melting peaks for PIB and VPI.

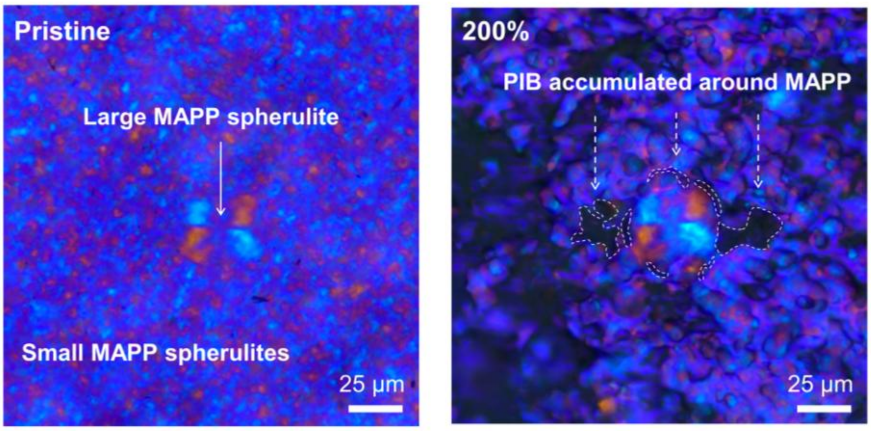

Supplementary Figure 4. Polarized optical micrographs of the VPI sample before and after 200% strain.

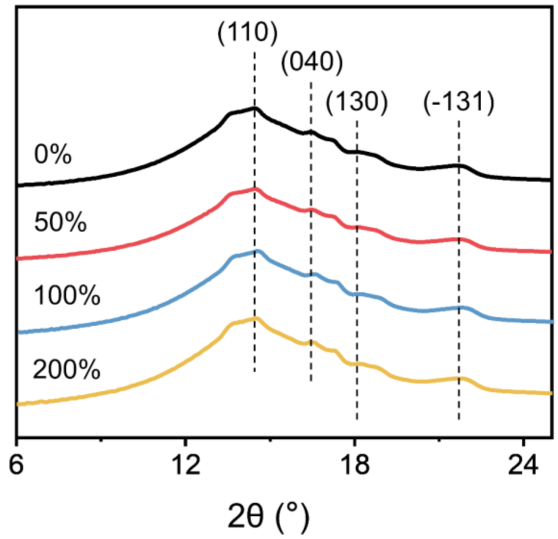

Supplementary Figure 5. WAXS curves of the VPI film after stretching to the predefined strains (0%, 50%, 100%, and 200%) at a strain rate of 0.5% s^{-1}.

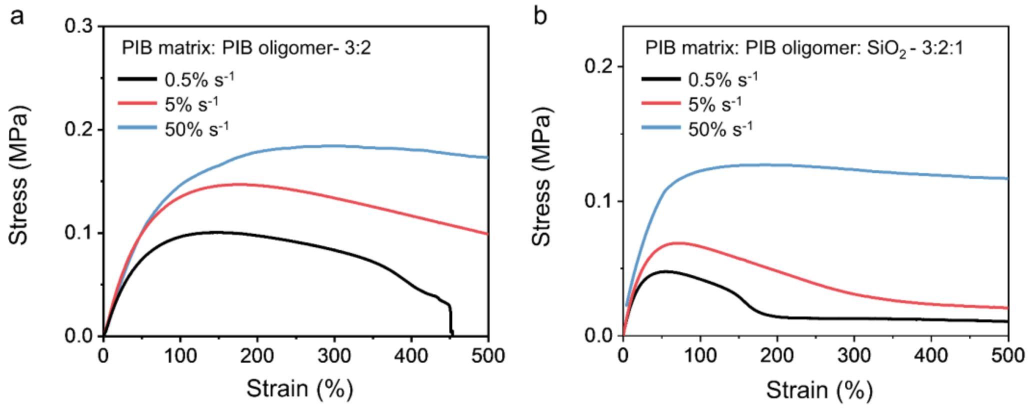

Supplementary Figure 6. Uniaxial stress-strain curves for different strain rates of (a) PIB matrix/PIB oligomer (3:2) and (b) PIB matrix: PIB oligomer: SiO_2 (3:2:1).

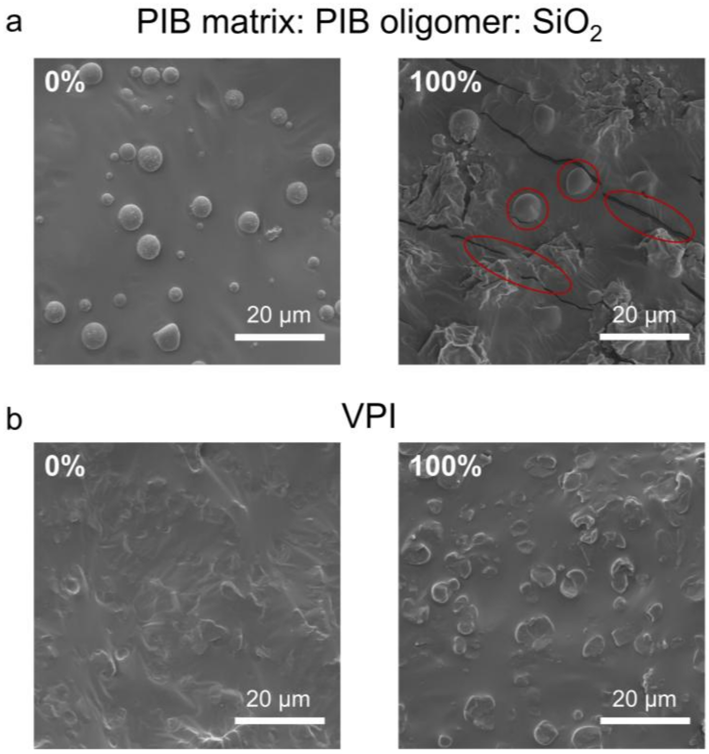

Supplementary Figure 7. The cross-sectional SEM images of (a) PIB matrix/PIB oligomer/SiO_2 (3:2:1) and (b) PIB matrix/PIB oligomer/MAPP (3:2:1) at 0% and 100% strain.

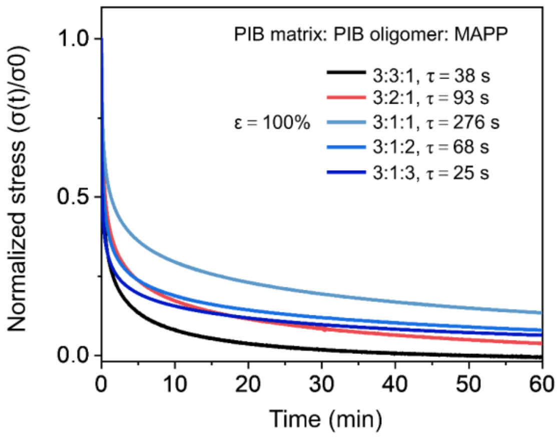

Supplementary Figure 8. Stress curves as a function of time at 100% strain under room temperature for the VPI films with different compositions.

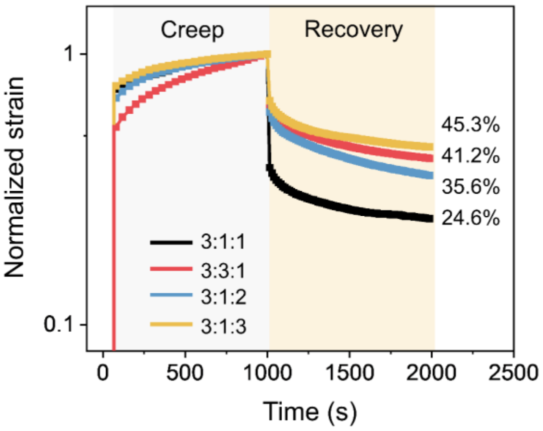

Supplementary Figure 9. Creep recovery test under constant shear stress (500 Pa) for 1000 s and release for 1000 s of the VPI with different mass ratios of high MW PIB, low MW PIB, and MAPP (3:1:1, 3:3:1, 3:1:2, and 3:1:3).

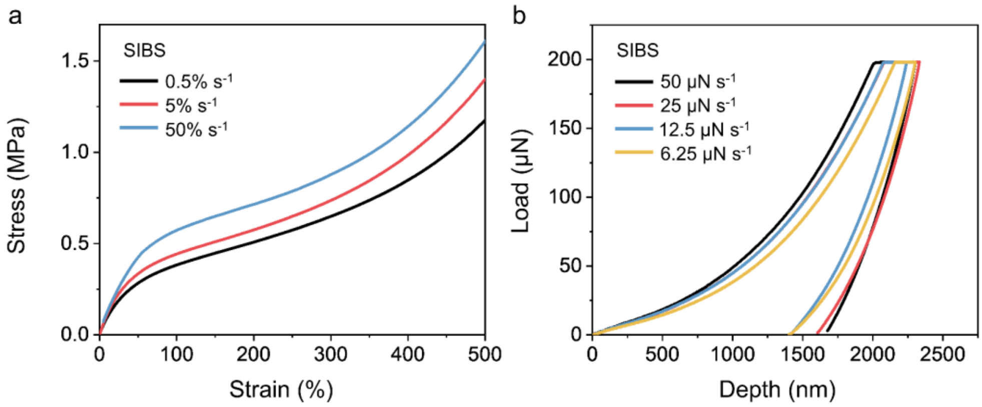

Supplementary Figure 10. (a) Uniaxial stress-strain curves for different strain rates of SIBS and (b) the typical load-displacement curves of SIBS at different loading rates from 6.25 to 50 μN s^{-1}.

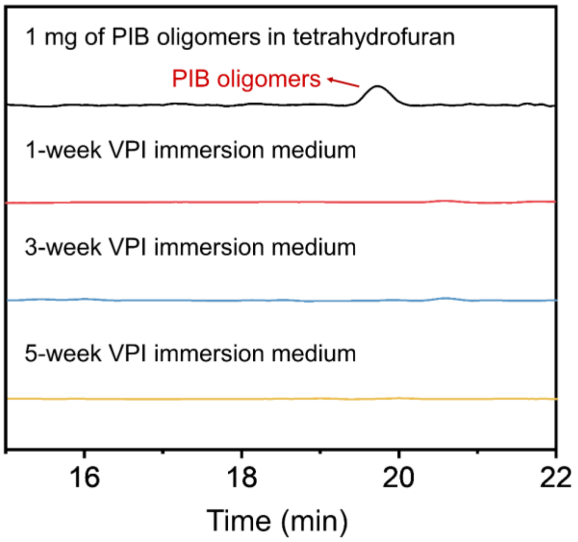

Supplementary Figure 11. Gas chromatography of VPI after being immersed in aqueous solutions at 37°C for 5 weeks. 1 mg of PIB oligomer was completely dissolved in 1 mL of tetrahydrofuran to prepare a standard sample.

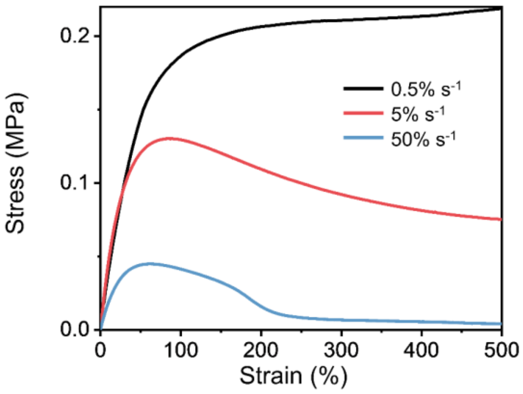

Supplementary Figure 12. Uniaxial stress-strain curves for different strain rates of VPI after being immersed in PBS solutions (pH ~7.4, 37°C) for 5 weeks.

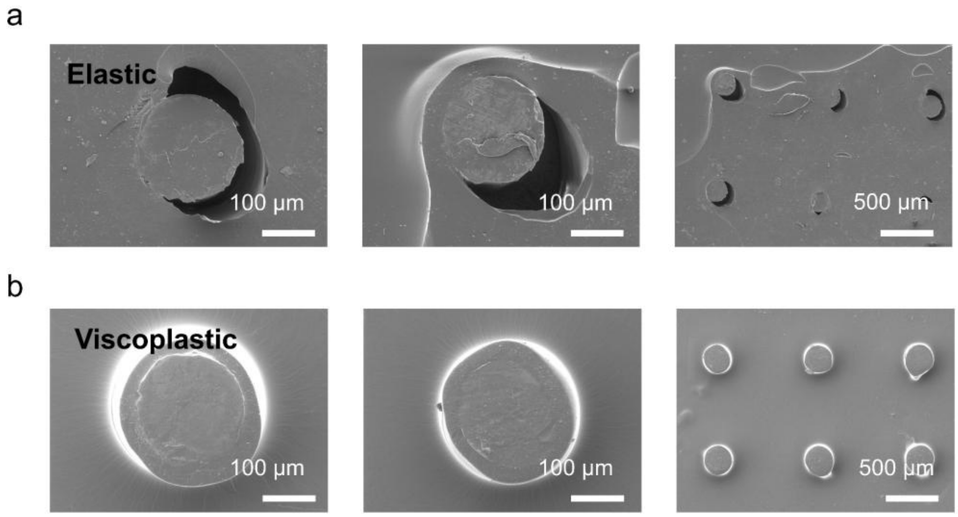

Supplementary Figure 13. Top-view SEM images of Cu electrode encapsulation, showing distinct interfacial gaps of (a) the elastic group and a void-free interface of (b) the viscoplastic group.

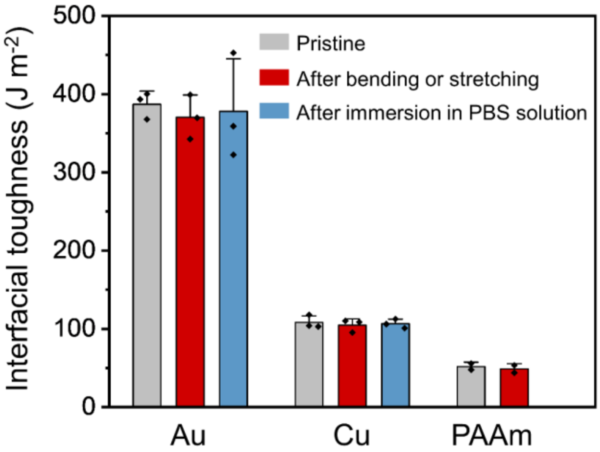

Supplementary Figure 14. The interfacial toughness of Au, Cu, and PAAm electrodes under different operating conditions. The conditions of cyclic mechanical strain testing were as follows: Au (bending radius of 10 mm, 1000 cycles, 1 Hz), Cu (bending radius of 5 mm, 10,000 cycles, 1 Hz), and PAAm (tensile strain of 30%, 10,000 cycles, 1 Hz). The Au and Cu electrodes were immersed in PBS (pH ~7.4, 37°C) for 2 weeks before the peel testing.

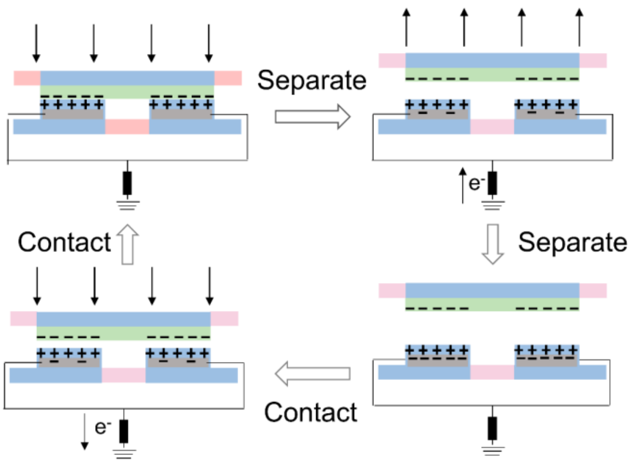

Supplementary Figure 15. Working principle of the device under a contact-separation mode based on triboelectric and electrostatic effects.

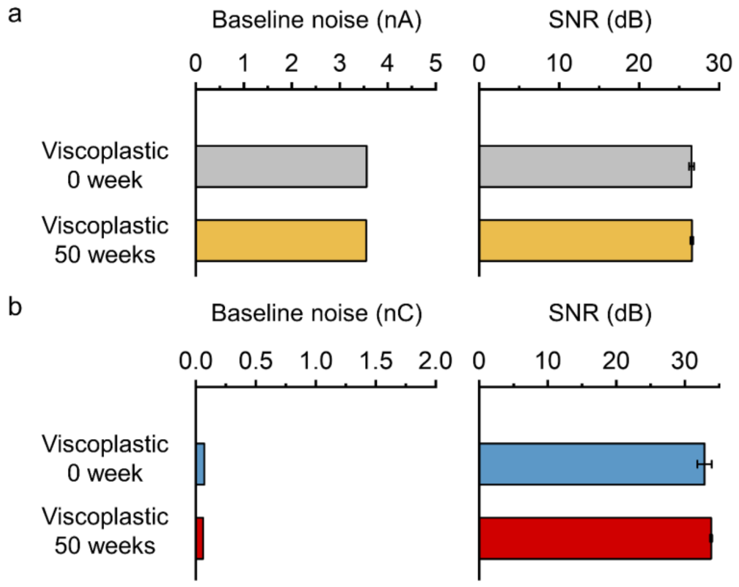

Supplementary Figure 16. Quantitative analysis of baseline noise (left) and SNR (right) for (a) current output and (b) charge output of the viscoplastic groups after immersing in PBS solution at room temperature.

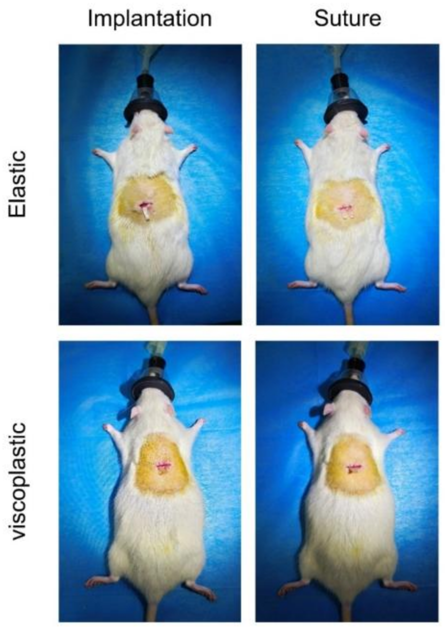

Supplementary Figure 17. Representative photos of subcutaneous implantation of the bioelectronic device in rats for the elastic and viscoplastic group. Surgical procedures involved creating a 15 mm cutaneous incision. The device was then placed between the epithelial and deep muscle layers, and the incision was finally sutured.

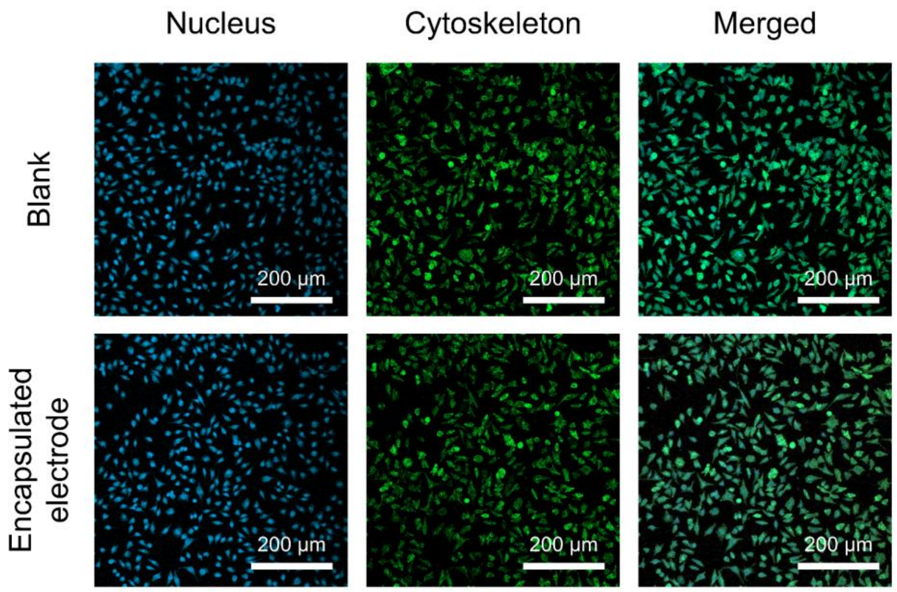

Supplementary Figure 18. Confocal fluorescence micrographs for the morphology of the L929 cells with stained cell nuclei (DAPI, blue) and stained cytoskeleton (FITC phalloidin, green) on the blank sample and encapsulated electrode after 24 h of culture.

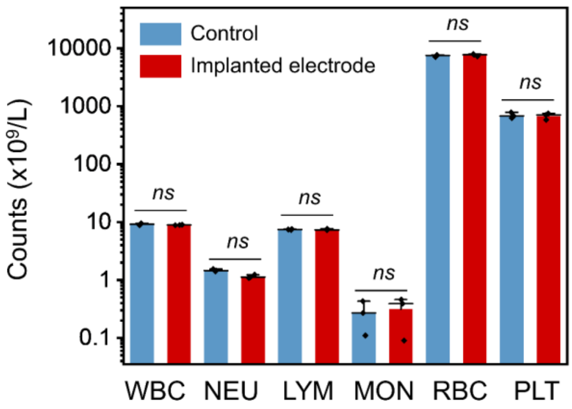

Supplementary Figure 19. Analysis of complete blood count (CBC) for control (healthy rats) and rats with the device implanted in the subcutaneous region on the back at 20 weeks. Independent samples, n = 3. Error bars, SD.

Table S1. Comparison of the *in vivo* recoding stability of implantable electrodes

	Encapsulation	Signal-to-noise ratio	Electrode	Function	Stable recording time	Ref.
**Plastics**	SU-8	8 dB	Pt	Neural signal	12 weeks	1
	Parylene C	10 dB	Carbon nanotube	Neural signal	10 weeks	2
	Parylene C	~5 dB	PEDOT-PSS, Au	Electromyography signal	1 week	3
	PVDF, PMMA	30 dB	Pt, Au, Cu	Neural signal	10 weeks	4
	PPy, Heparin	38.3 dB	Au	Electrocardiogram signal	10 days	5
	PI	30 dB	GO	Neural signal	12 weeks	6
**Elastomers**	SEBS	~15 dB	Au	Neural signal	43 weeks	7
	PDMS-MPU-IU	~25 dB	LM	Electrocardiogram signal	4 weeks	8
	PDMS	~10 dB	PEDOT: PSS	Neural signal	3 weeks	9
	PDMS	12.1 dB	Ag	Electromechanical signal	11 days	10
	PLCL-PLGA	~11 dB	Mo	Neural signal	2 weeks	11
	PLGA, PVA	~37dB	PEDOT-PSS, Au	Neural signal	6 days	12
	SEBS	20 dB	Au	Electromyography signal	3 weeks	13
	SBS	10 dB	PEDOT-PSS, Au	Neural signal	16 weeks	14
	VPI, SIBS	10 dB	Au	Electromechanical signal	45 weeks	This work

Abbreviations: Polyvinylidene fluoride (PVDF); Polymethyl methacrylate (PMMA); Styrene-ethylene-butylene-styrene (SEBS); Styrene-butadiene-styrene (SBS); Poly(lactide-co-ε-caprolactone)–poly(lactic-co-glycolic acid) (PLCL-PLGA); Polypyrrole (PPy); Polydimethylsiloxane (PDMS); Poly(lactic-co-glycolic acid) (PLGA); Poly(vinyl alcohol) (PVA); Polyimide (PI); Poly(3,4-ethylenedioxythiophene) polystyrene sulfonate (PEDOT-PSS); Liquid metal (LM); Graphene oxide (GO); Viscoplastic interlayer (VPI); Poly(styrene-block-isobutylene-block-styrene) (SIBS)

REFERENCES

1. Yang X, Zhou T, Zwang TJ *et al.* Bioinspired neuron-like electronics. *Nat Mater* 2019; **18**: 510–7.
2. McCallum GA, Sui XH, Qiu C *et al.* Chronic interfacing with the autonomic nervous system using carbon nanotube (CNT) yarn electrodes. *Sci Rep* 2017; **7**: 11723.
3. Boys AJ, Carnicer-Lombarte A, Gueemes-Gonzalez A *et al.* 3D bioelectronics with a remodellable matrix for long-term tissue integration and recording. *Adv Mater* 2023; **35**: 2207847.
4. Du MH, Huang L, Zheng JJ *et al.* Flexible fiber probe for efficient neural stimulation and detection. *Adv Sci* 2020; **7**: 2001410.
5. Lee S, Park J, Kim S *et al.* High-performance implantable bioelectrodes with immunocompatible topography for modulation of macrophage responses. *Acs Nano* 2022; **16**: 7471–85.
6. Viana D, Walston ST, Masvidal-Codina E *et al.* Nanoporous graphene-based thin-film microelectrodes for in vivo high-resolution neural recording and stimulation. *Nat Nanotechnol* 2024; **19**: 514–23.
7. Xie RJ, Han F, Yu QHY *et al.* A movable long-term implantable soft microfibre for dynamic bioelectronics. *Nature* 2025; **645**: 648-55.
8. Choi H, Kim Y, Kim S *et al.* Adhesive bioelectronics for sutureless epicardial interfacing. *Nat Electron* 2023; **6**: 779–89.
9. Won D, Kim H, Kim J *et al.* Laser-induced wet stability and adhesion of pure conducting polymer hydrogels. *Nat Electron* 2024; **7**: 475–86.
10. Chen H, Wang AQ, Chen J *et al.* Drug-screening triboelectric nanogenerator based strain sensor for cardiomyocyte contractility. *Nano Energy* 2023; **112**: 108251.
11. Bae JY, Hwang GS, Kim YS *et al.* A biodegradable and self-deployable electronic tent electrode for brain cortex interfacing. *Nat Electron* 2024; **7**: 815–28.
12. Wu MG, Yao KM, Huang NG *et al.* Ultrathin, Soft, Bioresorbable organic electrochemical transistors for transient spatiotemporal mapping of brain activity. *Adv Sci* 2023; **10**: 2300504.
13. Yi JQ, Zou GJ, Huang JP *et al.* Water-responsive supercontractile polymer films for bioelectronic interfaces. *Nature* 2023; **624**: 295–302.
14. Khatib M, Zhao ET, Wei SY *et al.* High-density soft bioelectronic fibres for multimodal sensing and stimulation. *Nature* 2025; **645**: 656–64.
